# Altered voxel-level whole-brain functional connectivity in multiple system atrophy patients with depression symptoms

**DOI:** 10.1186/s12888-022-03893-4

**Published:** 2022-04-20

**Authors:** Hua Guang Yang, Weiyin Vivian Liu, Zhi Wen, Lan Hua Hu, Guo Guang Fan, Yun Fei Zha

**Affiliations:** 1 Department of Radiology, Renmin Hospital of Wuhan University, Wuhan, 430060 China; 2 MR Research, GE Healthcare, Beijing, 100176 China; 3 Department of Radiology, The First Affiliated Hospital of China Medical University, Shenyang, LN China

**Keywords:** Multiple system atrophy, Depression symptoms, Functional magnetic resonance imaging, Degree centrality, Functional connectivity

## Abstract

**Background:**

It is yet unknown if the whole-brain resting-state network is altered in multiple system atrophy with symptoms of depression. This study aimed to investigate if and how depression symptoms in multiple system atrophy are associated with resting-state network dysfunction.

**Methods:**

We assessed the resting-state functional network matric using Degree centrality (DC) coupling with a second ROI-wise functional connectivity (FC) algorithm in a multimodal imaging case-control study that enrolled 32 multiple system atrophy patients with depression symptoms (MSA-D), 30 multiple system atrophy patients without depression symptoms (MSA-ND), and 34 healthy controls (HC).

**Results:**

Compared to HC, MSA-D showed more extensive DC hub dysfunction in the left precentral and right middle frontal cortex than MSA-ND. A direct comparison between MSA-D and MSA-ND detected increased DC in the right anterior cingulum cortex, but decreased DC in the left cerebellum lobule IV and lobule V, left middle pole temporal cortex, and right superior frontal cortex. Only right anterior cingulum cortex mean DC values showed a positive correlation with depression severity, and used ACC as seed, a second ROI-wise functional connectivity further revealed MSA-D patients showed decreased connectivity between the ACC and right thalamus and right middle temporal gyrus (MTG).

**Conclusions:**

These findings revealed that dysfunction of rACC, right middle temporal lobe and right thalamus involved in depressed MSA. Our study might help to the understanding of the neuropathological mechanism of depression in MSA.

**Supplementary Information:**

The online version contains supplementary material available at 10.1186/s12888-022-03893-4.

## Background

Multiple system atrophy (MSA) is a rare and progressive neurodegenerative disease characterized by prominent non-motor system symptoms and autonomic dysfunction symptoms [1]. Depression is one of the most common and frequently underestimated neuropsychiatric conditions in multiple system atrophy (MSA). A recent epidemiological study found that about 70% of patients with newly developed MSA manifested depression symptoms [2]. Importantly, depression may occur at any stage during MSA and worsen the motor and other non-motor symptoms of MSA patients [3]. At present, the mechanism of MSA with depressive symptoms is unclear, which makes the clinical treatment mainly focus on the anti-depression treatment similar to the pure depression diseases. However, different from depression disease, the neurotransmitter disorder in MSA patients with depressive symptoms injury mainly destroys dopaminergic neurons that were supplemented by serotonin, norepinephrine and other neurotransmitter dysfunction [4]. Therefore, digging out the depression-modulated pathogenesis in the MSA is helpful to treatment selection. However, the neuropathological mechanisms underlying depressive MSA and non-depressive MSA are yet to be deciphered.

Functional magnetic resonance imaging (fMRI) is an established technique that potentially provided useful insights into the neuropathological mechanisms of depression. Previous studies using fMRI and single-photon emission computerized tomography (SPECT) have revealed that depression symptoms in MSA are linked to the abnormalities of anatomy, connectivity, and metabolism in frontal, limbic, and motor networks [5, 6]. For example, the amygdala is a functional hub for adverse emotions in the brain, and, as a seed of functional connectivity (FC), a recent study showed that the depression symptoms manifested by MSA patients were associated with amygdala-frontal and occipital FC decreased [5]. Similarly, a SPECT study observed that the severity of depression was significantly associated with the metabolism of dorsolateral prefrontal glucose in MSA patients with depression symptoms [6], suggesting that a cortex network dysfunction may contribute to depression symptoms in MSA patients. Because only a few neuroimaging studies have focused on depression in MSA patients, limiting a solid understanding of depression-related network alterations, any inferences currently made should be assimilated with caution.

Degree centrality (DC) is a pure data-driven resting state function MRI research method, which avoids the bias caused by seed selection. This graph-based measurement of network organization reflects the number of instantaneous functional connections between a region and the rest of the brain within the entire connectivity matrix of the brain [7]. Thus, it is the most direct analytical method in description of the centrality of a node in the network as the impact and function of the nodes. Previous functional MRI studies used DC maps have identified functional network complexity patterns in several neuropsychiatric diseases [8–10]. In this research, we hypothesized that depression stems from alterations in the resting-state function network of MSA patients, which can be untangled using DC method. We first used the degree centrality method to assess depression-driven cerebral hub alterations in MSA-D. Using these different hubs as seeds, we further investigated how hub-dominated function network alterations contribute to depression process in MSA.

## Subjects and methods

### Participants

From February 2014 to January 2020, 32 (16 females, 16 males) MSA-D and 30 multiple system atrophy patients without depression symptoms (MSA-ND) (14 females, 16 males) patients were registered for this study by in the Department of Neurology, Renmin Hospital, Wuhan University and the First Affiliated Hospital of China Medical University. 34 healthy controls with matched age, gender, and education also enrolled from the same city through advertisements between 2014 and 2020 (control participants have been used in our previous analyses [11, 12]).

Enrolled MSA patients satisfied the following inclusion criteria: (1) Diagnosed with MSA by a movement disorder specialist according to the MSA diagnostic criteria (second edition, 2008) as either probable or possible MSA [13]; (2) The onset of depression symptoms occurred after the incidence of the MSA disease. Exclusion criteria were: (1) Cognitive impairment (Mini-mental State Examination (MMSE) score < 26); (2) Current or previous use of antipsychotic drugs; (3) history of neurological diseases or any predominant physical diseases.

Demographic data including gender, age, educational years, subtype, disease duration, and treatment drugs of the patients were collected by neurologists in face-to-face interviews. The Unified Multiple System Atrophy Rating Scale (UMSARS) and ﻿Hoehn and Yahr (H-Y) stage were used to evaluate motor severity. Levodopa Equivalent dose (LEDD) was used to evaluate the drug use in MSA patients. Mini-Mental State Examination (MMSE) was used to evaluate cognitive impairment. Severity of depression and anxiety were evaluated using the Hamilton depression (HAMD) and anxiety (HAMA) scales, ﻿respectively Sixty-two MSA patients were divided into two groups of 32 MSA-D and 30 MSA-ND according to the fifth edition of the Diagnostic and Statistical Manual of Mental Disorders (DSM-V) [14]. The severity of depression symptoms was evaluated per the 24 terms Hamilton Depression Scale (HAMD-24). All subjects were told about the purpose of the study and signed informed consent before enrollment. Our study was approved by the ethics committee of both hospitals.

### MRI data acquisitions and preprocessing

Participant MRI data were acquired with GE MR750W 3 Tesla scanners (GE Healthcare, Milwaukee, WI, USA) located at the Department of Radiology, People’s Hospital of Wuhan University, and at the First Affiliated Hospital of China Medical University. A body transmit and eight-channel receive head coil were used. The total MRI scan time was 15 min and 47 s, which included a regular clinical sequence (to discharge brain diseases), a high-resolution three-dimensional sagittal magnetization prepared gradient echo imaging sequence (3D-T1), and a blood oxygen level-dependent (BOLD) sequence scan. The parameters of the BOLD sequence were as follows: repetition time = 2000 ms, echo time = 25 ms, flip angle = 90, slice number = 40, slice thickness = 3 mm without slice gap, field of view [FOV] = 240 mm × 240 mm, matrix size = 64 × 64, and voxel size = 3 mm × 3 mm × 3 mm. The 3D-T1 sequence had the following parameters: repetition time/echo time = 8.5/3.3, matrix = 256 × 256, flip angle =12°,voxel size = 1.0 mm × 1.0 mm × 1.0 mm, slice thickness = 1 mm without slice gap, FOV = 256 × 256 mm^2^, and slice number = 180.

Resting-state functional MRI data preprocessing analyses were performed using the Data Processing & Analysis for Brain Imaging (DPABI) toolbox (version 3.0, www.restfmri.net). The detailed preprocessing steps included: (1) disregarding the first 10 time points; (2) slice timing; (3) volume realignment (Subjects with head movement greater than 2.5 mm or 2.5 degrees in any direction were excluded from the present study); (4) regression of nuisance covariates (Friston-24 parameters were applied, subjects’ white matter and cerebrospinal fluid signals were also removed) [15]; (5) normalization (gray matter were normalized to Montreal Neurological Institute template); (6) detrending; (7) band-pass filtering (0.01–0.08 Hz). Any volume with a frame-wise displacement value exceeding 0.5 mm were scrubbed to exclude from the present study [45].

### Degree centrality

Each voxel of the brain can be regarded as a node, and the DC of a node refers to the sum of direct connections between itself and other voxels as functional connectivity between nodes. The larger the node degree of a node, the higher the degree centrality of the node and the more important the node is to the network.

For DC calculation, the DPABI software was used to analyze the preprocessed fMRI data, and the correlations between the time series of each voxel and those of all voxels were used to create the DC map of the whole brain as the connection matrix of the gray matter of the whole brain. We obtained an n × n Pearson’s correlation matrix, where n represents the voxel number of the whole gray matter (GM) mask. The correlation value was further transformed with Fisher Z to meet normal distribution. *r* > 0.25 was selected as the threshold value to screen out the correlation value, and the whole-brain function network was established [11, 16]. A 6 mm × 6 mm × 6 mm full width at half maximum (FWHM) Gaussian kernel was used to smooth the maps (the data preprocessing was without smoothing). Only positive weighted Pearson correlation coefficients were considered as the number of functional connections at the individual level because of the uncertainty in interpreting negative values.

### Functional connectivity

The preprocessed image was further smoothed with a 6 mm^3^ Gaussian kernel for the calculation of functional connectivity (the image is not smoothed during DC calculation). For DC analysis, we selected DC results with significant relationships to the HAMD-24 scale between MSA-D and MSA-ND as seeds to detect alterations of brain resting-state brain network linked to depression symptoms in MSA patients. Specifically, we extracted the reference time series from DC results by averaging the time series of every voxel in seed regions and conducted further correlation analyses between the time series of each voxel inside and outside of the seed regions in the entire brain. The correlation coefficients were then converted into Z values using Fisher’s r-to-z transformation. Additionally, we analyzed the FC map of the spherical region within a 3 mm radius that covers the peak group difference between DC values to eliminate seed selection-related influence (See supplementary materials 1).

### Statistical analysis

#### Statistical analysis of clinical data

The SPSS 22.0 software (SPSS Inc., Chicago, Illinois, USA) was used to statistically analyze demographic and clinical data. Data were first tested for normal distribution in relation to clinical data assumption. The independent sample t-test and Kruskal-Wallis test or ANOVA (followed by Tukey’s test for normally distributed data or the Bonferroni test for non-normally distributed data) were used for cross-group comparisons of quantitative variables. The χ^2^ test was used to compare qualitative variables. We set the significance threshold at *p* < 0.05 for all analyses.

#### Statistical analysis of neuroimaging data

The DPABI statistical analysis module was used for Neuroimaging Data analysis. To explore DC differences among the MSA-D, MSA-ND, and HC groups, we performed one-way ANOVA with age, sex, MMSE, and unified multiple system atrophy rating scale (UMSARS) scores as covariates. Next, we conducted post-hoc analyses to assess the differences between groups within the masks with significant ANOVA results and used similar covariates with ANOVA for multiple comparison corrections (AlphaSim correction, *P* < 0.001) We performed a voxel-wise Pearson correlation analysis of depression scale scores obtained in the assessment of patients with MSA-D to identify brain regions that significantly associated with clinical depression symptoms in MSA patients.

To determine FC network alterations in patients with MSA-D, brain regions showing significant depression-related in DC were selected as seeds for a secondary seed-based FC analysis. FC post-hoc analyses between groups shared similar covariates with ANOVA Furthermore, we analyzed the correlation between depression and whole-brain seed-based RSFC at the voxel level.

## Results

### Clinical and neuropsychological characteristics

The demographic and clinical characteristics of the MSA-D, MSA-ND, and HC groups are shown in Table 1. There were no significant differences in age, gender, education, and MMSE score among all three groups. There were also no significant differences in the course of the disease, the UMSARS score, clinical subtypes, and the H-Y stage between the MSA-D group and the MSA-ND group. However, the HAMD-24 score of the MSA-D group was significantly higher than that of the MSA-ND group.

**Table 1:** Demographic and clinical characteristics

Characteristics (Mean ± SD)	Control (*n* = 34)	MSA-D (*n* = 32)	MSA-ND (*n* = 30)	F/χ2	*P*-value
Age (years)	63.97 ± 5.08	63.03 ± 5.94	64.70 ± 7.74	0.55	0.58
Gender (male: female)	20:24	16:16	16:14	0.26	0.77
Education	11.44 ± 3.11	11.00 ± 4.31	11.27 ± 3.61	0.12	0.89
Disease duration	N	3.17 ± 1.63	3.57 ± 1.83	0.48	0.49
UMSARS score	N	33.22 ± 17.47	28.05 ± 18.63	0.05	0.83
Hoehn and Yahr	N	2.53 ± 1.09	2.37 ± 1.16	0.14	0.71
LEDD (mg/day)	N	335.94 ± 292.10	330.00 ± 354.31	0.94	0.17
Clinical phenotype (P/C)	N	15:17	13:17	0.28	0.60
MMSE score	28.78 ± 1.35	28.00 ± 1.78	28.40 ± 1.04	2.37	0.58
HAMD-24 score	1.5 ± 1.81	20.00 ± 6.25	4.80 ± 2.44	196.59	0.00*
HAMA-14 score	1.15 ± 1.10	3.44 ± 1.52	3.23 ± 1.71	24.98	0.00*
Dopamine agonists	N	20.16 ± 35.01	33.33 ± 76.38	2.61	0.11

*SD* Standard deviation, *MSA* Multiple system atrophy, *MSA-D* Multiple system atrophy with depression symptoms, *MSA-ND* Multiple system atrophy without depression symptoms, *HC* Healthy controls, *UMSARS* Unified Multiple System Atrophy Rating Scale, *LEDD* Levodopa Equivalent dose, *P/C* Parkinson’s type/Cerebellar type, *MMSE* Mini-Mental State Examination, *HAMD-24* 24 items Hamilton Depression Scale. *p* < 0.05 was considered statistically significant. *:*p*<0.001.HAMA-14: 14 items Hamilton Anxiety Scale

### DC analysis

**Fig. 1:**
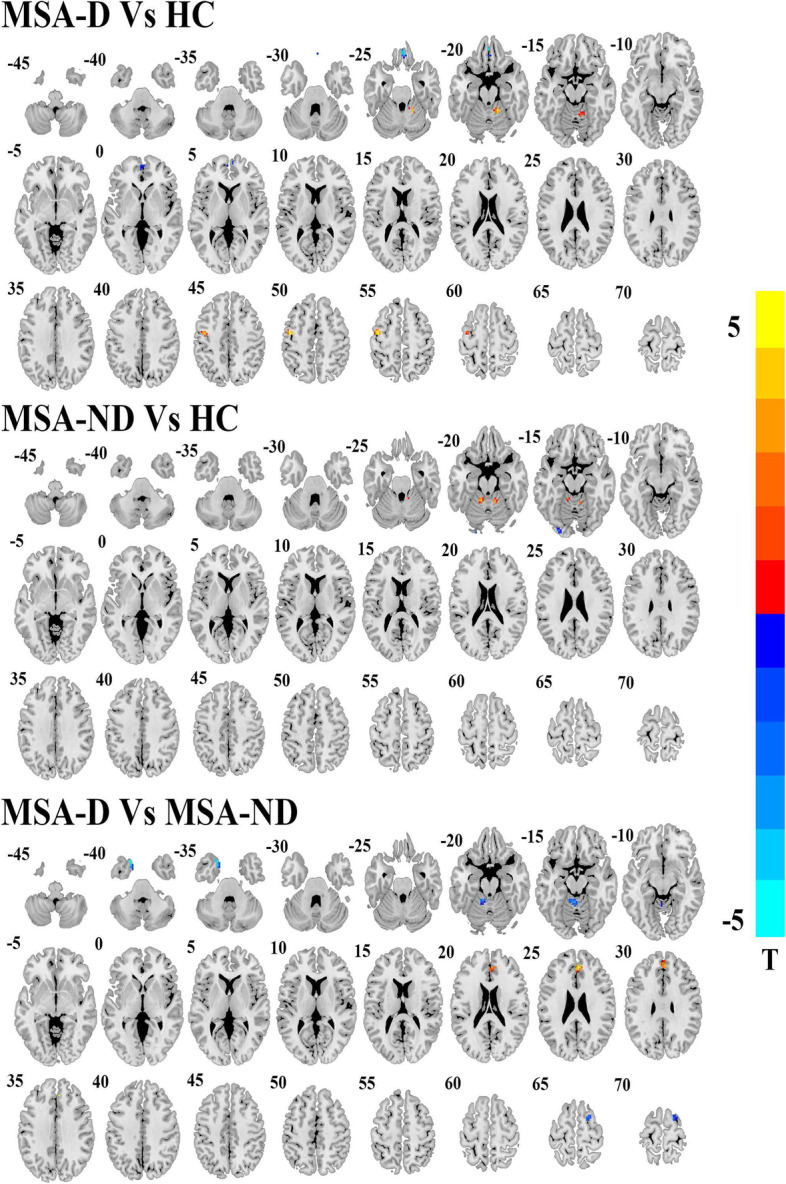
Brain regions show degree centrality differences among depressed MSA, non-depressed MSA and HCs. The threshold value was set as *P* < 0.001(AlphaSim multiple comparisons corrected). MSA-D: multiple system atrophy with depression symptoms; MSA-ND: multiple system atrophy without depression symptoms; HC: healthy controls

Compared to HC, MSA-D had increased DC in the right Table 2 and left precentral gyrus but decreased DC in the left rectus and right middle frontal cortex. MSA-ND had increased DC in the bilateral cerebellum lobule IV and lobule V and decreased DC in the left lingual gyrus. Compared to the MSA-ND group, MSA-D showed increased DC in the right anterior cingulate cortex (ACC) and decreased DC in the left cerebellum lobule IV and lobule V, left middle pole temporal cortex, and right superior frontal cortex (Table 2, Fig. 1).

**Table 2:** Brain regions showing differences in degree values between MSA patients and HC

Brain Regions	Hem	Voxels	BA	Peak MNI Coordinate			T Value
				x	y	z	
**MSA-D Vs HC**							
Rectus gyrus (aal)	L	21	NA	0	42	-24	−4.83
Cerebellum_4_5(aal)	R	35	30	15	−45	−21	4.93
Middle frontal gyrus (aal)	R	22	10	6	60	3	−3.98
Precentral (aal)	L	51	6	−42	−6	54	5.11
**MSA-ND Vs HC**							
Cerebellum_4_5(aal)	R	18	19	9	−48	−18	4.19
Lingual (aal)	L	17	18	−27	−87	−18	−4.16
Cerebellum_4_5(aal)	L	22	30	−12	−42	− 18	4.53
**MSA-D Vs MSA-ND**							
Middle pole temporal gyrus (aal)	L	23	36	−24	15	−39	−5.25
Cerebellum_4_5(aal)	L	60	NA	−3	−45	−18	−4.53
Anterior Cingulum cortex (aal)	R	46	32	3	42	27	4.60
Superior frontal gyrus (aal)	R	23	6	21	3	66	−4.02

A negative T value represents a decreased degree in the MSA group. *MSA-D, MSA-ND* Multiple system atrophy patients with and without depression symptoms. *BA* Brodmann area, *L, R* Left and Right, *Hem* Hemisphere

### Seed-based FC analysis

**Fig. 2:**
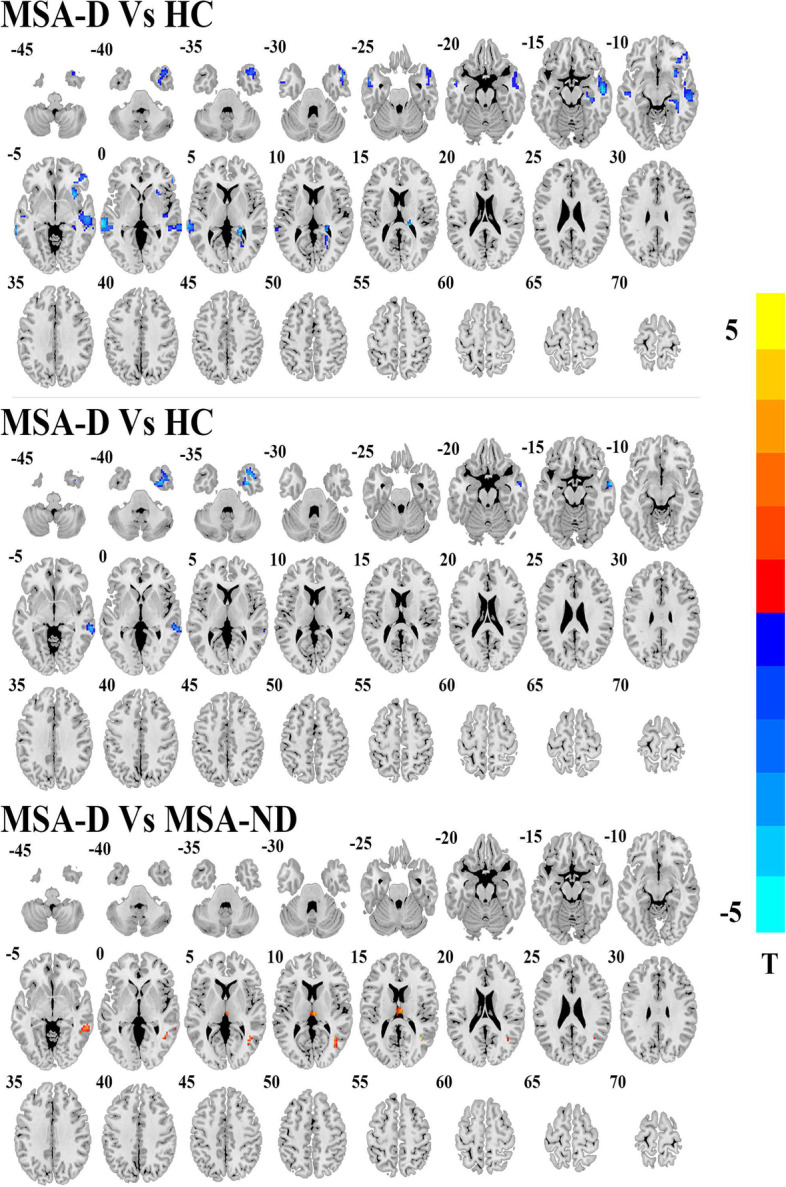
Post hoc two-sample t-test results of right ACC seed FC analyses differences among MSA-D, MSA-ND and HCs. AlphaSim multiple comparisons corrected *p* < 0.001

**Fig. 3:**
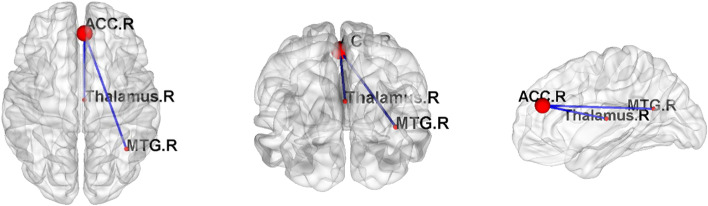
Post hoc two-sample t-test results of right ACC seed FC analyses between MSA-D and MSA-ND groups. The graph of the network is made by BrainNet (https://www.nitrc.org/projects/bnv/) software. Red node represents the node of brain area, and the blue edge represents the decrease of functional connection. R, right

Compared with HC, MSA-D showed decreased ACC-based FC in the bilateral MTG, right hippocampus, right insula, right inferior frontal cortex and right calcarine, whereas, MSA-ND showed decreased ACC-based FC in the inferior and middle temporal gyrus. Direct comparison of the MSA-D and MSA-ND groups revealed ACC-based FC in the right thalamus, right MTG, and right middle cingulum cortex (Table 3, Figs. 2 and 3).

**Table 3:** Brain regions showing right ACC-related FC network alterations between MSA patients and HC

Brain Regions	Hem	Voxels	BA	Peak MNI Coordinate			T Value
				x	y	z	
**MSA-D Vs HC**							
Middle temporal gyrus (aal)	R	391	20	54	−9	−15	−5.70
Middle temporal gyrus (aal)	L	129	21	−69	−30	0	−5.95
Hippocampus (aal)	R	51	20	27	−27	−12	−4.83
Insula (aal)	R	44	NA	33	15	−6	−5.06
Inferior frontal gyrus (aal)	R	39	45	54	39	0	−4.51
Calcarine (aal)	R	59	37	24	−39	6	−5.61
**MSA-ND Vs HC**							
Inferior temporal gyrus (aal)	R	72	36	33	0	−39	−3.83
Middle temporal gyrus (aal)	R	57	21	63	−33	− 3	−3.86
**MSA-D Vs MSA-ND**							
Middle temporal gyrus (aal)	R	80	39	39	−57	15	4.50
Thalamus (aal)	R	45	48	3	−15	15	−4.84

A negative T value represents a decreased right ACC-related FC in the MSA group. *MSA-D, MSA-ND* Multiple system atrophy patients with and without depression symptoms, *BA* Brodmann area, *L, R* Left and Right, *Hem* Hemisphere

### Correlation between DC and seed-based FC changes with depression scores in the MSA group

The severity of depression symptoms was reflected by the HAMD-24 scale.

**Fig. 4:**
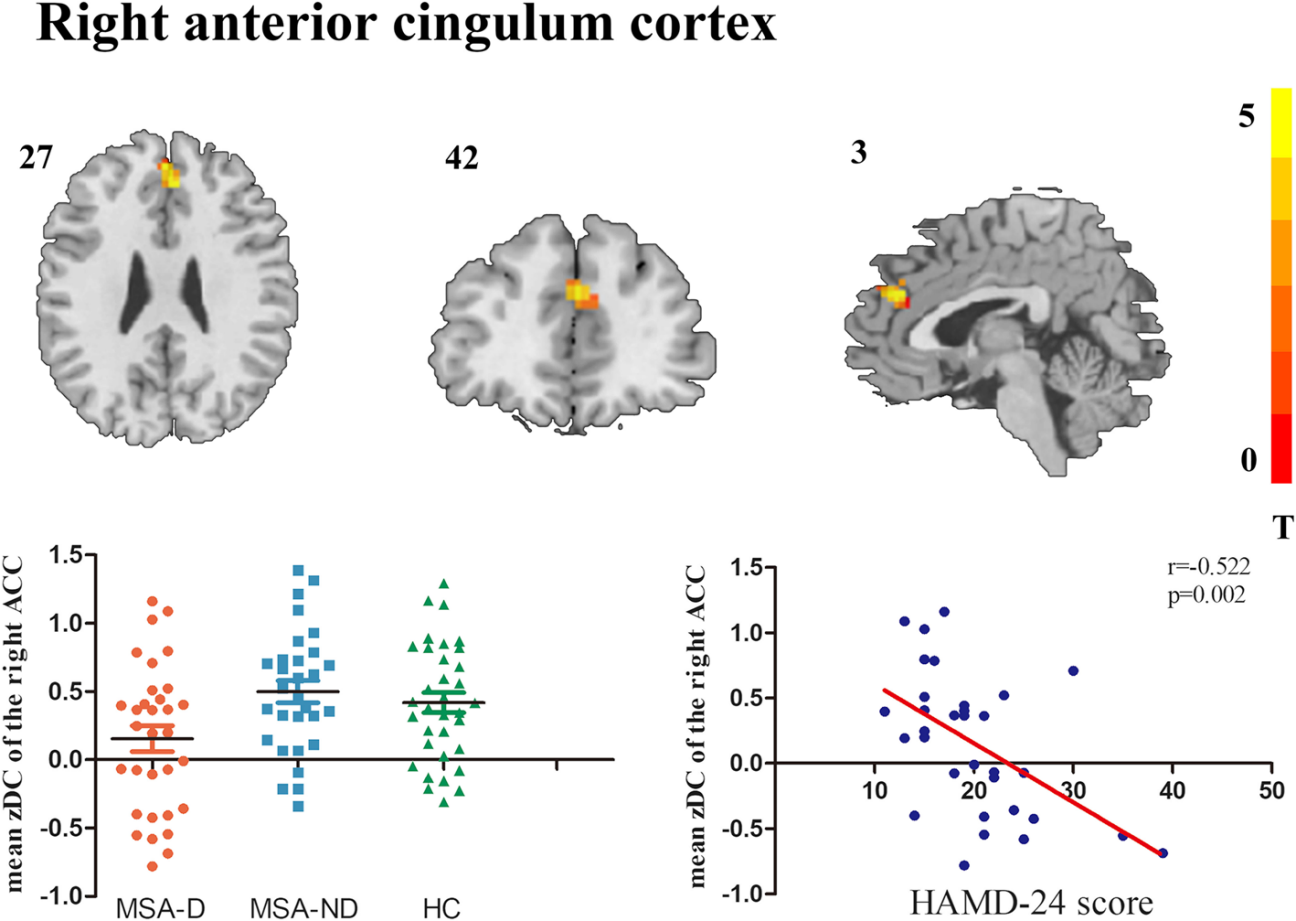
Compared with MSA-ND group, the right ACC was the only brain area show DC alteration correlated with clinical depression scores. Scatter plot showed a negative correlation between HAMD-24 scores and the right ACC zDC values in the MSA-D patients

A voxel-wise Pearson correlation analysis was used to identify brain regions significantly associated with clinical depression symptoms in MSA patients, and only the right ACC DC alteration was found to be significantly associate with HAMD-24 scores (Fig. 4). Figure 4 shows the findings of the correlation analysis between DC mapping and HAMD-24 values in patients with MSA (Fig. 4).

## Discussion

Depression is a common non-motor system symptom in patients with MSA. Understanding of the neuroimaging mechanism underpinning the depression symptoms in MSA could realize the possibility of the therapeutic effect. By analysis of degree centrality and secondary functional connectivity, we found that MSA patients with depression symptoms harbored changes in the resting-state network of the cortex and subcortical whole-brain. In our first analysis, the right ACC was the only significant brain area involved in the depression symptom process in MSA patients. Second seed-based FC analysis showed that functional network changes between right ACC to the right thalamus and right MTG may be reckoned as neuropathological hubs underlying depressed MSA These results complement previous research on abnormalities of the whole-brain resting-state functional network in MSA patients with depression symptoms.

Compared with MSA-ND, MSA-D showed increased activation in the right ACC. As an important node of the default mode network (DMN), previous studies have found that abnormal ACC mainly contributes to cognitive impairment [18]. In the present study, increased DC in the right ACC was correlated negatively with HAMD-24 scores after excluding cognitive influences. In fact, such a result is not necessarily contradictory. The functioning of the ACC was not only associated with cognitive control but also with emotion regulation-related process; the ACC is one of the most consistently recurring brain regions in patients with major depressive disorder [19, 20]. Evidence from voxel-based morphometry studies has shown that decreased gray matter volume in the ACC is associated with depression severity in patients with major depressive disorder patients, whereas an increased in the ACC is the primary reason for the improvement on negative emotional stimuli under an electroconvulsive therapy [21, 22], suggesting that ACC plays a key role in the depression process. Only one study reported that the ACC’s involvement of ACC in regulation of depression symptoms in MSA patients. Using the amygdala as the seed, a recent resting-state FC study also established that the alteration in the amygdala-ACC network was associated with depression symptoms in MSA patients [6], Therefore, consistent with previous studies, our results showed the ACC as the key cortical pathogenesis involved in the depression process of MSA patients. Additionally, we speculated that the positive association of the zDC value in the right ACC and HAMD-24 scores may reflect a compensatory mechanism for counteracting functional or structural brain abnormalities. More MSA patients with different depression levels must be studied to satisfactorily soothe our theories.

A second seed-based FC revealed that MSA-D patients show FC decreased between ACC and right MTG, but FC increased between ACC and right thalamus. The thalamus is a complex information node, in organization of the sensory, motor, and cognitive processes and associated with specific cortical and subcortical regions [23]. Reduced thalamic gray matter volume was found in both clinical and subclinical MDD patients, indicating the thalamus is also involved in depression regulation [21, 24–26]. Other studies have reported that this outcome may stem from the connection between thalamus and the negative emotion-generating limbic structures, such as amygdala [27]. Therefore, abnormal DC in ACC, is coupled with thalamic functional connectivity network disorder and it, may account for the deficits in the top-down regulation of negative emotions among MSA patients who are more prone to experience depression symptoms. As a node of DMN, the MTG abnormalities might contribute to deficits in the “automatic” and controlled processing of emotional stimuli in patients with depression [28]. Functional and structural abnormalities in the DMN region are common in patients with depression [29–31] and also in Parkinson’s Disease patients with depression symptoms [32–34]. Therefore, the abnormality of the ACC and other subregions in DMN potentially constitutes the dysregulation of automatic emotional processing in MSA patients with depression symptoms. Of course, such a conclusion requires more different parts of DMN as seeds to be assessed in the study of MSA patients with depression.

“Aside from the above findings, compared with the HCs, the depressed MSA patients also showed extensive voxel-level whole brain DC/FC changes located in other brain areas, such as the cerebellum lobule IV and V, left precentral gyrus, middle temporal gyrus, right middle frontal cortex, hippocampus and insula. According to previous studies, abnormal structures such as right middle frontal cortex, hippocampus and insula were mainly involved in emotional regulation and dysfunction of these regions perhaps serves as a key neural network mechanism underlying depressed MSA [35, 36]. Both cerebellum lobule IV and V and precentral gyrus reflected the primary motor network impairment underlying MSA, However, recent studies have shown that the cerebellum is also involved in non-motor functions, including emotion regulation [37, 38]. We therefore believe that our findings further strengthen the theory that MSA-related dopamine depletion is not limited to the extrapyramidal system, but extends to other nonmotor systems where dopamine depletion that occurs may lead to some nonmotor symptoms, including depression.”

We observed that the brain regions associated with depression symptoms were located mainly in the right hemisphere of MSA patients. The right hemisphere is involved in negative emotions processing, pessimistic thoughts, and unconstructive thinking styles [39]. Many neuroimaging studies have shown that negative mood and depression are associated with relatively greater activity in the right hemisphere, compared to the homotopic region in the left hemisphere [40–42]. Therefore, it is not surprising that DC and secondary FC changed in the right hemisphere of patients with MSA. However, it should be noted that injured unilateral brain function network was commonly linked to asymmetries and specific symptoms in patients with MSA. Our previous study indicated that motor and cognitive impairments in MSA patients are largely linked to right lateral brain dysfunction [11, 12, 43], possibly because MSA patients predominantly have a right lateral onset as participants. Therefore, it must be confirmed that the depression symptoms of MSA patients are mainly associated with changes in the right lateral brain area network with further experiments under controlling the disease onset.

## Limitations

Firstly, in spite of 12 h washout before the scan and the equivalent doses of MSA-D and MSA-ND dopamine among enrolled patients, we could not exclude the residual medicine influence itself because the effect of levodopa on MSA is unknown and cannot be avoided [44]. Secondly, in terms of methodology referred in previous studies [11, 17, 45], only a threshold value *r* > 0.25 was used in our study when calculating DC, although we should have included more thresholds for comparisons to eliminate the influence of methodological choice on experimental results. Thirdly, MSA is a rare disease with an incidence rate of only 7/100000. More than 30 patients, it remained insufficient. Therefore, more patients should be recruited for future research to obtain more reliable results.

## Conclusion

MSA patients with depression mainly showed the changes of central hub and functional connectivity mainly occurred in temporal lobe and subcortical thalamic nuclei of MSA patients with depression compared to those only with MSA. In addition, the importance of rACC as a hub decreased and FC functioned worse in the right middle temporal lobe and right thalamus. The distinct network impairment between MSA patients with and without depression indicated different transmitter system dysfunctions were involved. This study provided neuroimaging evidence for a clinical understanding of non-pure depression and possibility of the therapeutic effect.

## Supplementary Information

**Additional file 1.**

**Additional file 2.**

## Data Availability

The datasets used or analyzed during the current study are available from the corresponding author on reasonable request.

## Abbreviations

MSA: Multiple system atrophy
MSA-ND: Multiple system atrophy patients with depression symptoms
MSA-ND: Multiple system atrophy patients without depression symptoms
HC: Healthy controls
DC: Degree centrality
FC: Functional connectivity
fMRI: Functional magnetic resonance imaging
SPECT: Single-photon emission computerized tomography
BOLD: Blood oxygen level-dependent
MMSE: Mini-mental State Examination
HAMD-24: 24 items Hamilton Depression Scale
UMSARS: Unified multiple system atrophy rating scale
MTG: Middle temporal gyrus
ACC: Anterior cingulum cortex
LEDD: Levodopa Equivalent dose
P/C: Parkinson’s type/Cerebellar type
BA: Brodmann area
L, R: Left and Right
DMN: Default mode network
