# Supplementary material for: Altered voxel-level whole-brain functional connectivity in multiple system atrophy patients with depression symptoms
Source: BMC Psychiatry. 2022 Apr 20;22:279. doi: 10.1186/s12888-022-03893-4 (PMC9020004; doi:10.1186/s12888-022-03893-4)
Supplement: Supplementary file 1 — Additional file 1. [file 12888_2022_3893_MOESM1_ESM.pptx]

## Slide 1
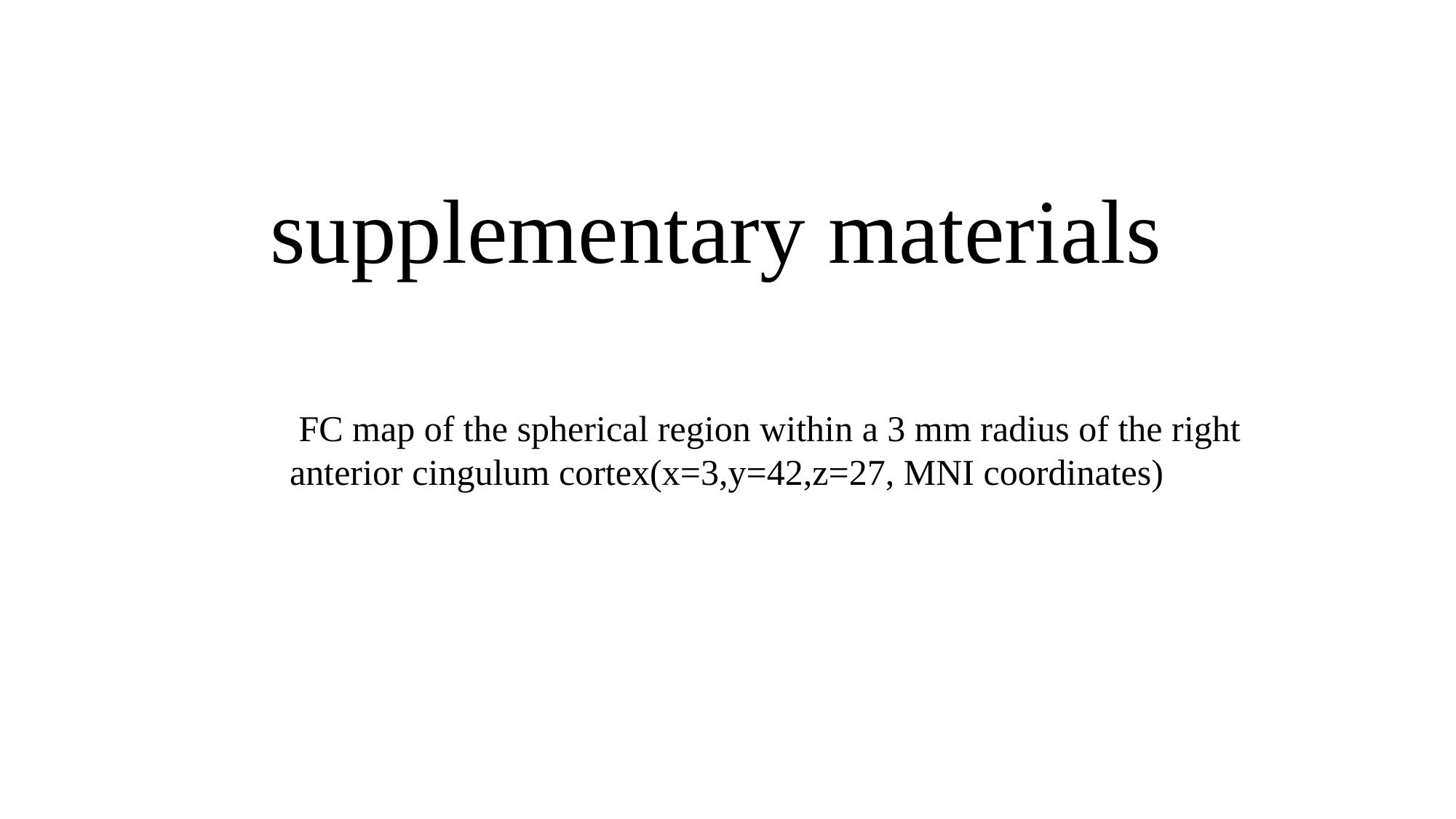

# supplementary materials
 FC map of the spherical region within a 3 mm radius of the right anterior cingulum cortex(x=3,y=42,z=27, MNI coordinates)

## Slide 2
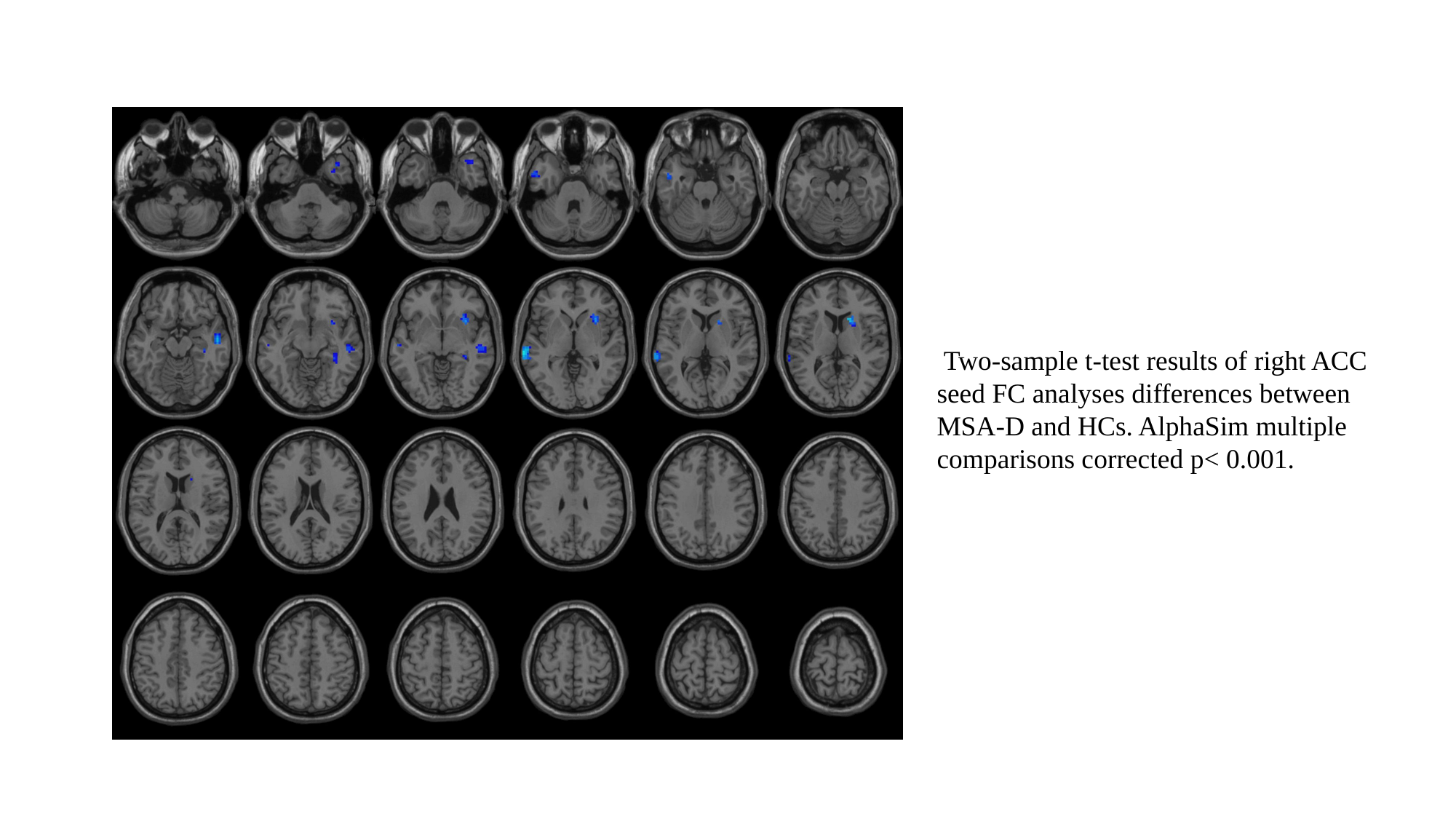

Two-sample t-test results of right ACC seed FC analyses differences between MSA-D and HCs. AlphaSim multiple comparisons corrected p< 0.001.

## Slide 3
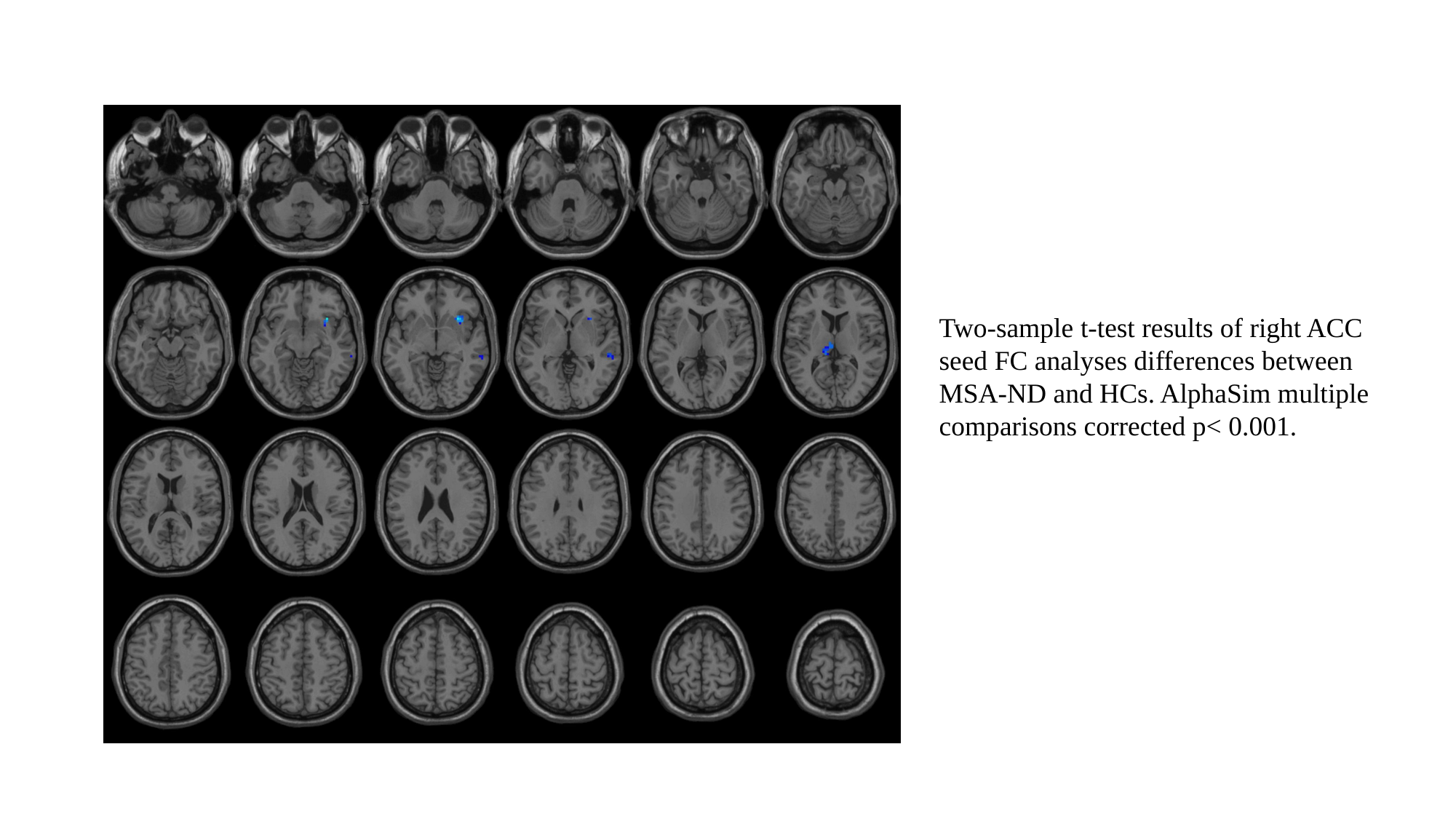

Two-sample t-test results of right ACC seed FC analyses differences between MSA-ND and HCs. AlphaSim multiple comparisons corrected p< 0.001.

## Slide 4
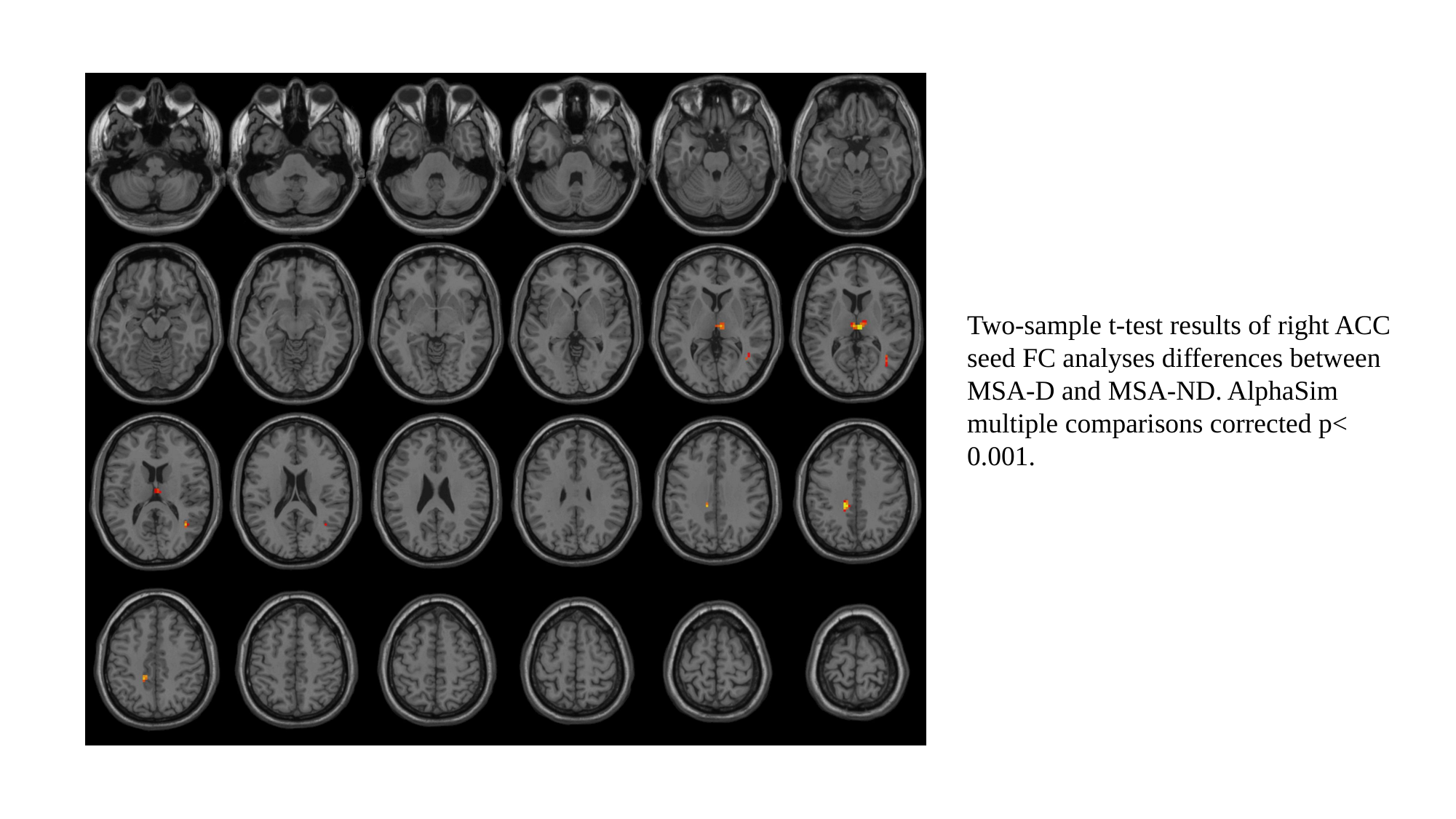

Two-sample t-test results of right ACC seed FC analyses differences between MSA-D and MSA-ND. AlphaSim multiple comparisons corrected p< 0.001.
