# Supplementary material for: Altered voxel-level whole-brain functional connectivity in multiple system atrophy patients with depression symptoms
Source: BMC Psychiatry. 2022 Apr 20;22:279. doi: 10.1186/s12888-022-03893-4 (PMC9020004; doi:10.1186/s12888-022-03893-4)
Supplement: Supplementary file 2 — Additional file 2. [file 12888_2022_3893_MOESM2_ESM.pptx]

## Slide 1
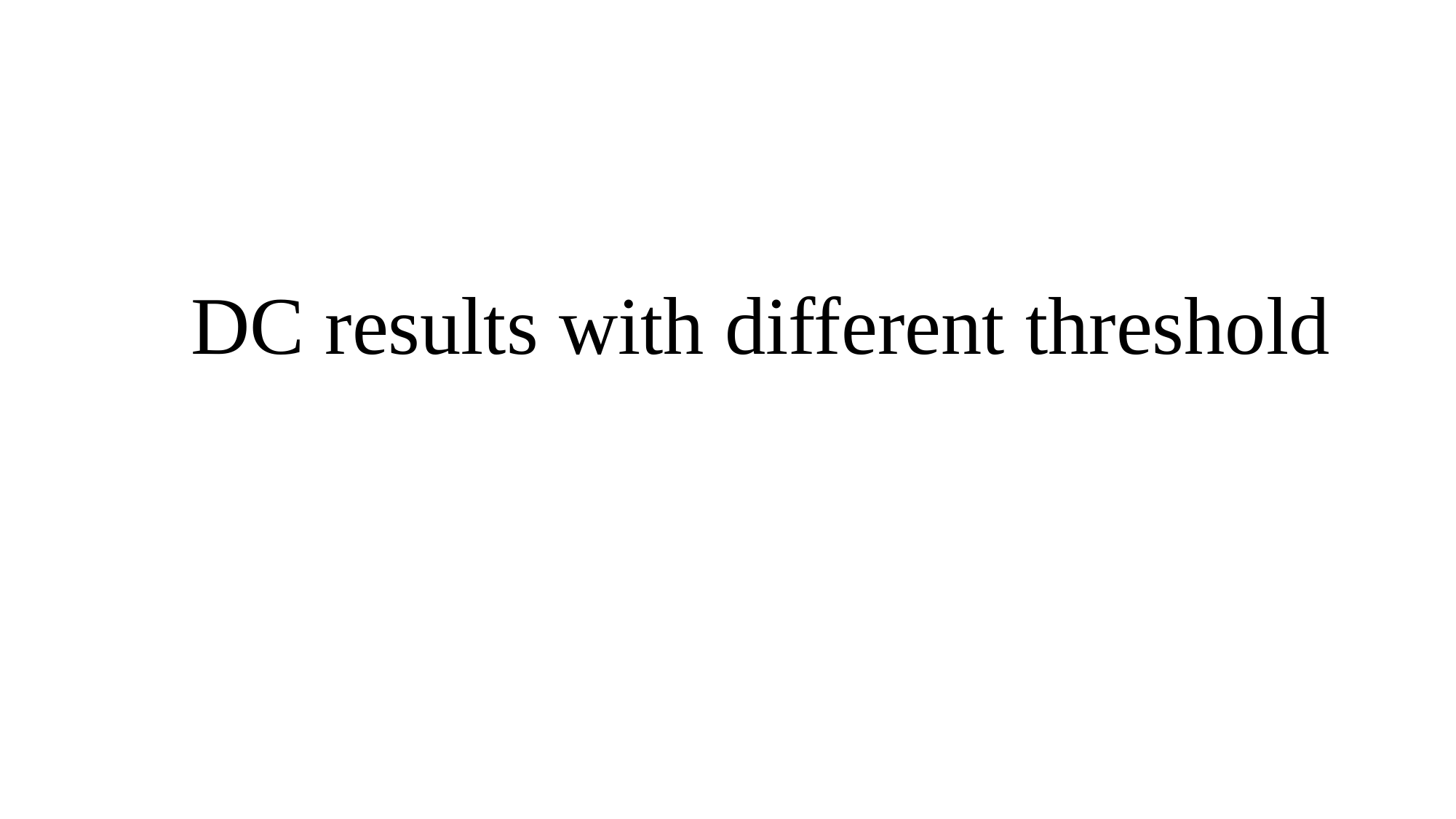

# DC results with different threshold

## Slide 2
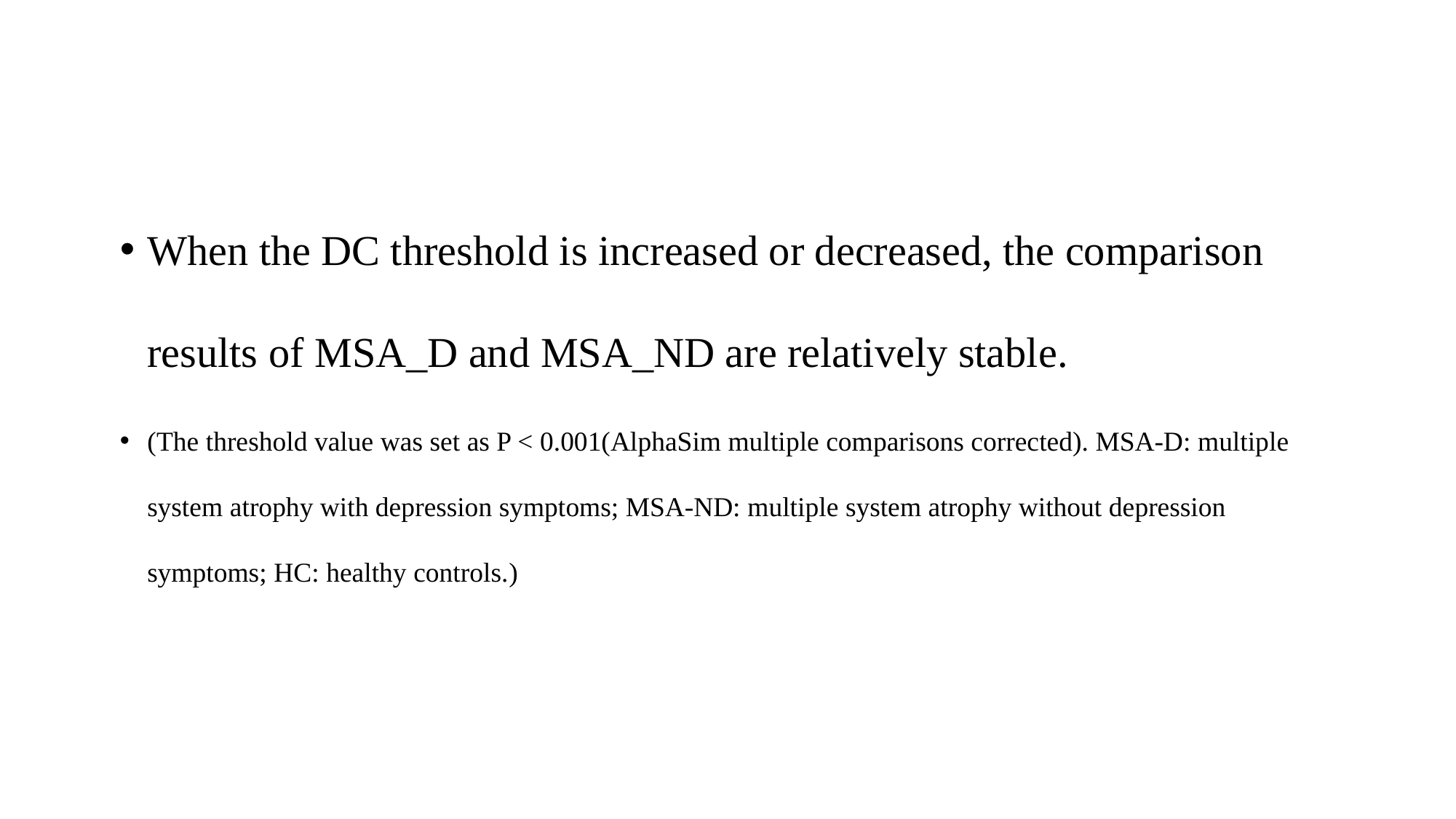

When the DC threshold is increased or decreased, the comparison results of MSA_D and MSA_ND are relatively stable.
(The threshold value was set as P < 0.001(AlphaSim multiple comparisons corrected). MSA-D: multiple system atrophy with depression symptoms; MSA-ND: multiple system atrophy without depression symptoms; HC: healthy controls.)

## Slide 3
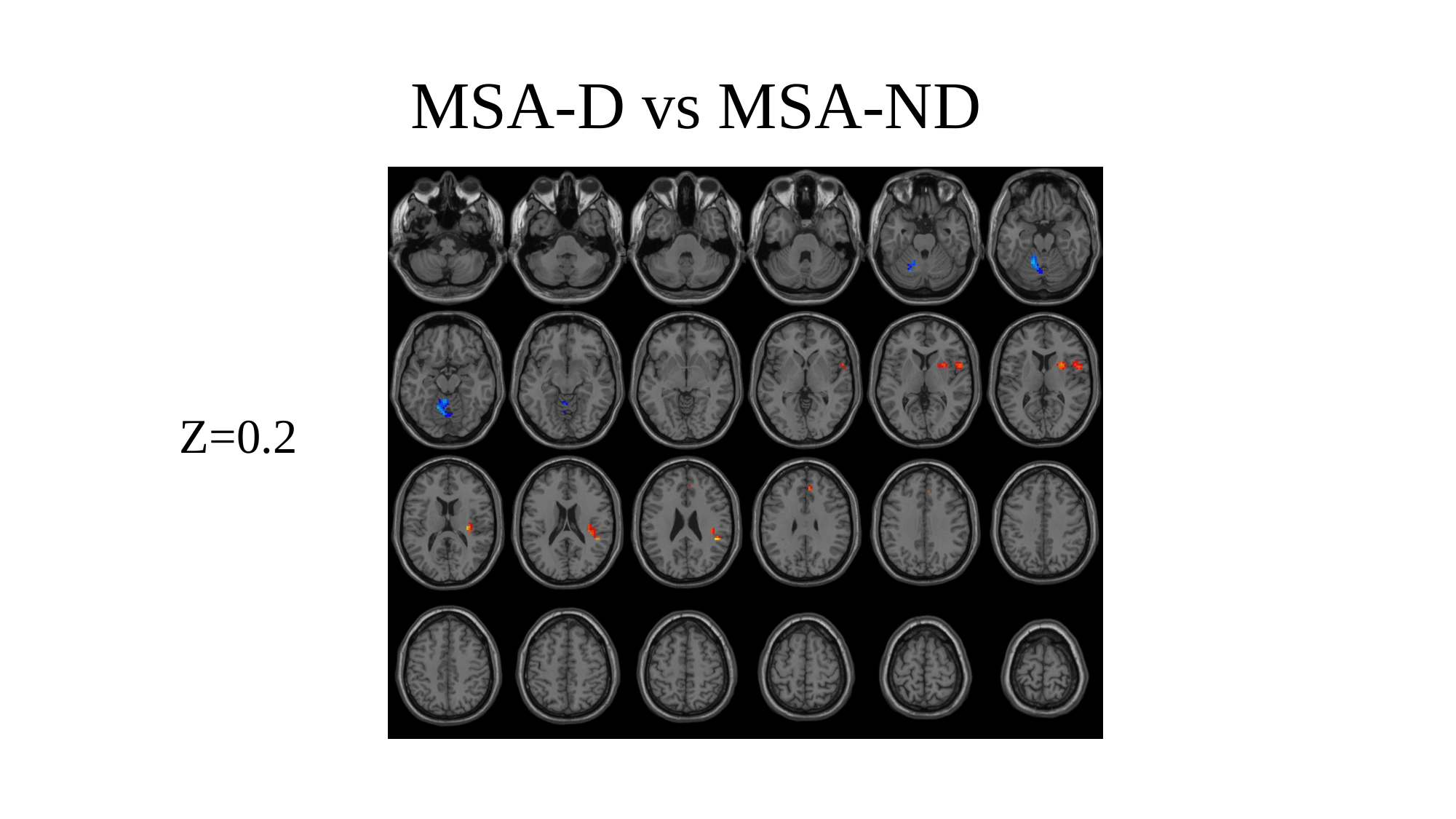

# MSA-D vs MSA-ND
Z=0.2

## Slide 4
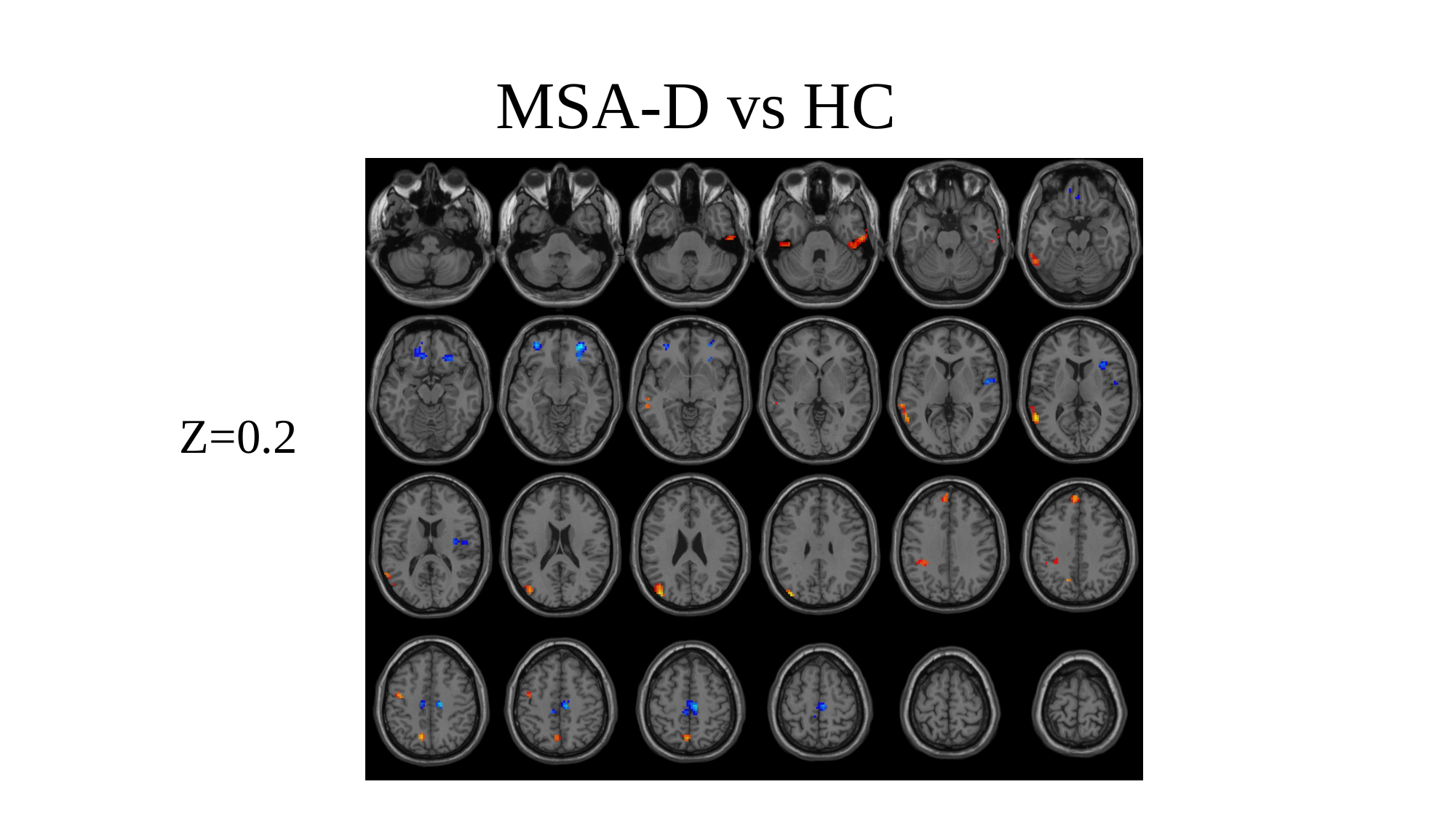

# MSA-D vs HC
Z=0.2

## Slide 5
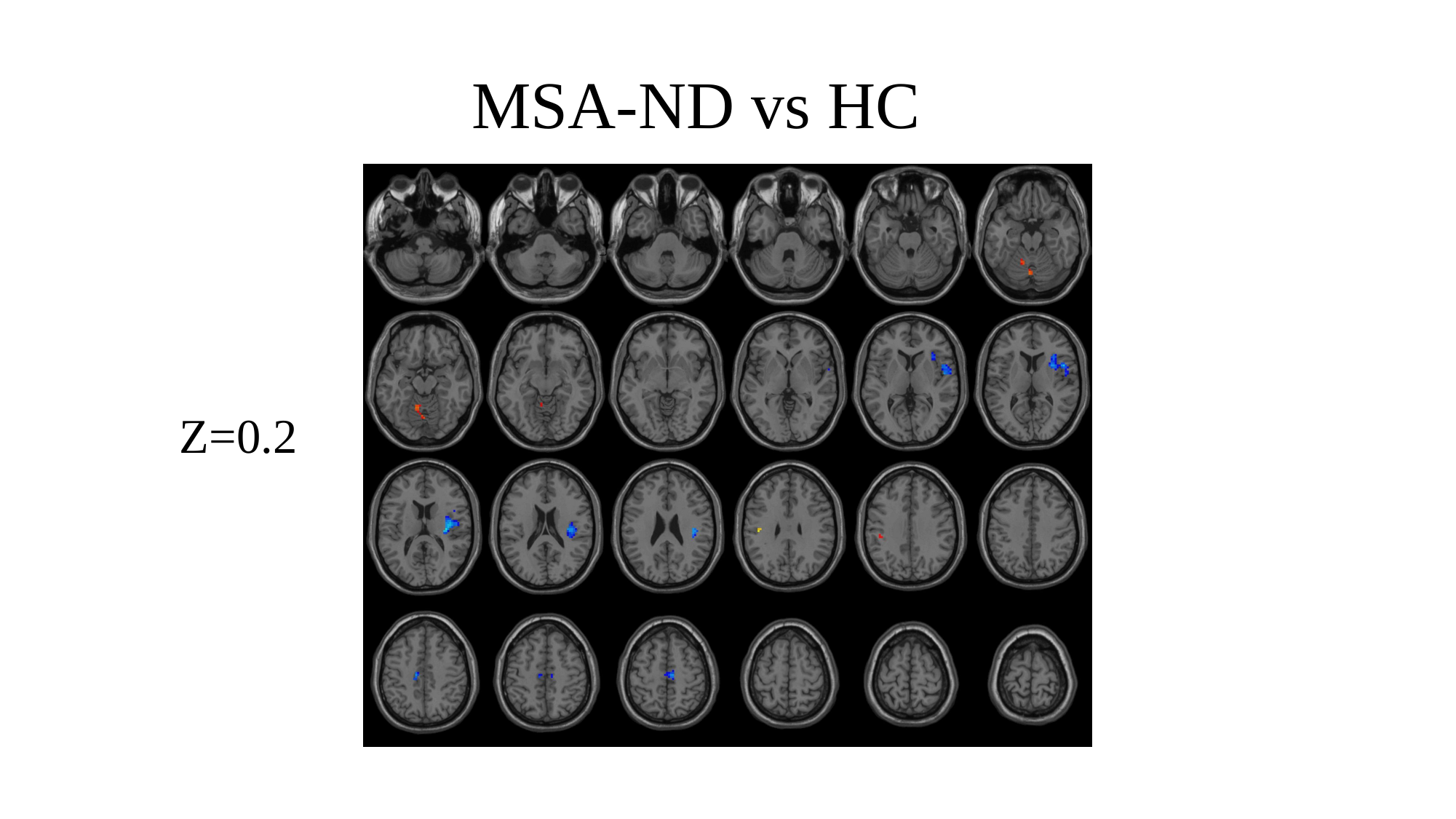

# MSA-ND vs HC
Z=0.2

## Slide 6
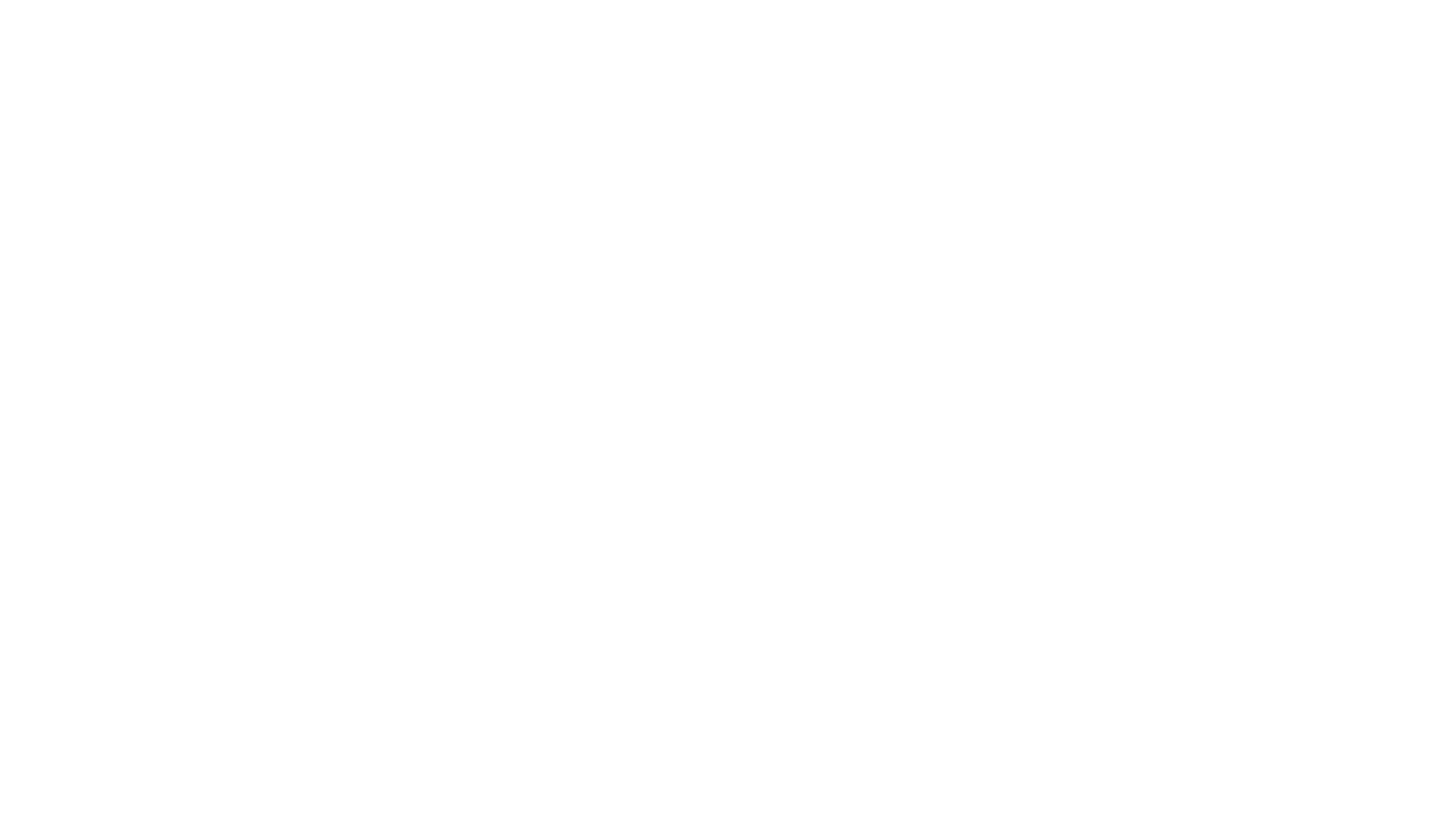

## Slide 7
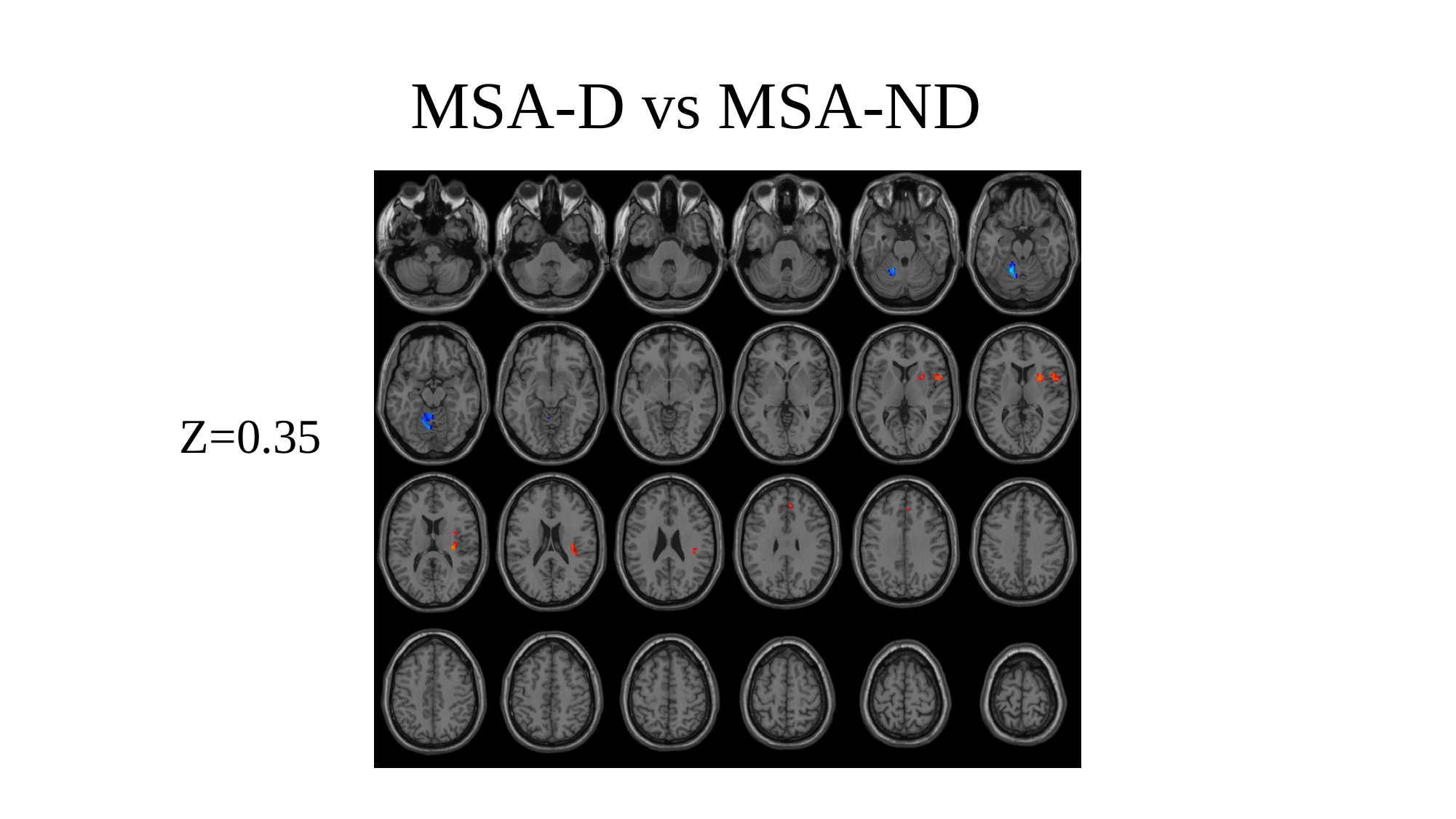

# MSA-D vs MSA-ND
Z=0.35

## Slide 8
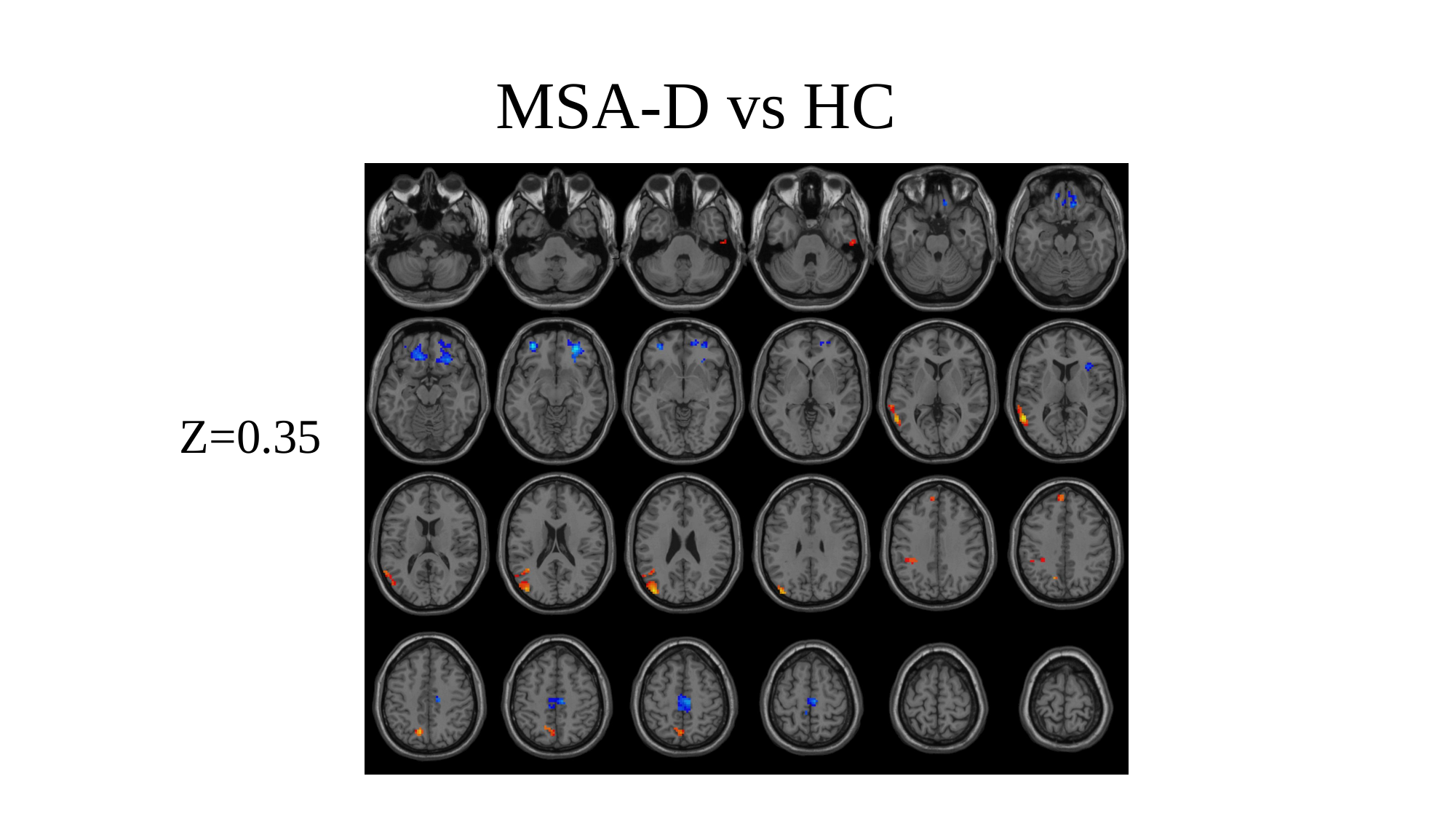

# MSA-D vs HC
Z=0.35

## Slide 9
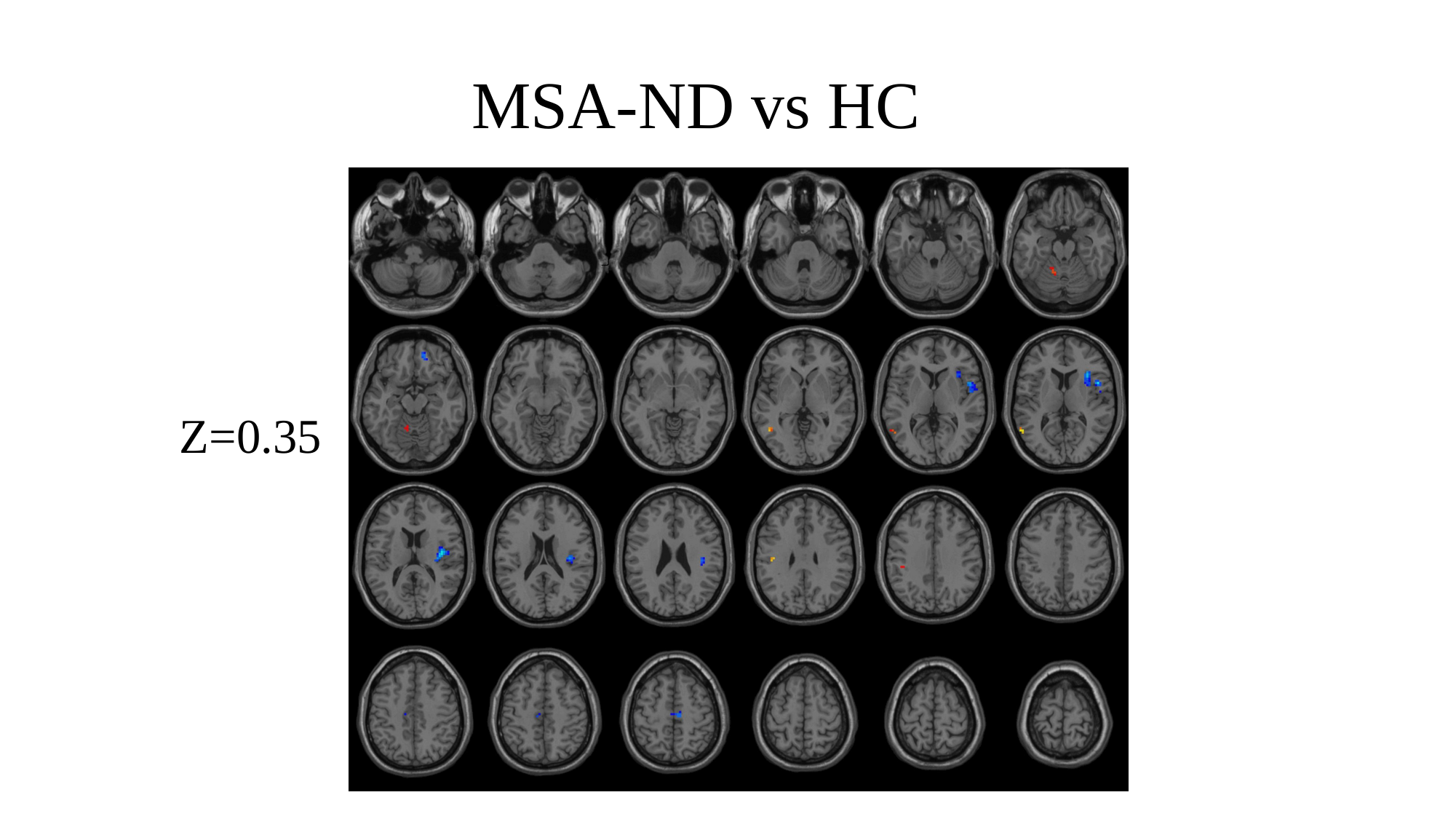

# MSA-ND vs HC
Z=0.35
